# Assessing the WEPP model performance for predicting daily runoff in three terrestrial ecosystems in western Syria

**DOI:** 10.1016/j.heliyon.2021.e06764

**Published:** 2021-04-24

**Authors:** Safwan Mohammed, Mais Hussien, Karam Alsafadi, Ali Mokhtar, Guido Rianna, Issa Kbibo, Mona Barkat, Swapan Talukdar, Szilárd Szabó, Endre Harsanyi

**Affiliations:** a Institution of Land Utilization, Technology and Regional Planning, University of Debrecen, 4032, Böszörményi út 138, Debrecen, Hungary; b Department of Soil Science, Faculty of Agriculture and Natural Resources, Tehran University, Tehran, Iran; c Department of Geography and GIS, Faculty of Arts, Alexandria University, Alexandria, Egypt; d Department of Agricultural Engineering, Faculty of Agriculture, Cairo University, Giza, 12613, Egypt; e State of Key Laboratory of Soil Erosion and Dryland Farming on Loess Plateau, Northwest University of Agriculture and Forestry, Institute of Soil and Water Conservation, Chinese Academy of Sciences and Ministry of Water Resources, Yangling, 712100, China; f Regional Models and Geo-Hydrological Impacts (REMHI) Division, Fondazione Centro Euro-Mediterraneo sui Cambiamenti Climatici, Via T. A. Edison, 81100, Caserta, Italy; g Department of Soil and Water Science, Faculty of Agriculture, Tishreen University, Lattakia, Syria; h Department of Geography, University of Gour Banga, Malda, 732101, India; i Department of Physical Geography and Geoinformatics, Faculty of Science and Technology, University of Debrecen, 4032, Debrecen, Hungary; j School of Geographical Sciences, Nanjing University of Information Science and Technology, Nanjing 210044, China

**Keywords:** Soil erosion, WEPP model, Runoff prediction, Ecosystem, Statistical analysis

## Abstract

Soil erosion is one of the main threats facing the agriculture and natural resources sector all over the world, and the same is true for Syria. Several empirical and physically based tools have been proposed to assess erosion induced soil losses and runoff driving the processes, from plot to regional spatial scales. The main goal of this research is to evaluate the performance of the Water Erosion Prediction Project (WEPP) model in predicting runoff in comparison with field experiments in the Al-Sabahia region of Western Syria in three ecosystems: agricultural lands (AG), burned forest (BF) and forest (FO). To achieve this, field experimental plots (2∗1.65∗0.5 m) were prepared to obtain runoff observation data between September 2012 and December 2013. In addition, the input data (atmospheric forcing, soil, slope, land management) were prepared to run the WEPP model to estimate the runoff. The results indicate that the average observed runoffs in the AG, BF and FO were 12.54 ± 1.17, 4.81 ± 0.97 and 1.72 ± 0.16 mm/event, respectively, while the simulated runoffs in the AG, BF and FO were 15.15 ± 0.89, 9.23 ± 1.48 and 2.61 ± 0.47mm/event, respectively. The statistical evaluation of the model's performance showed an unsatisfactory performance of the WEPP model for predicting the run-offs in the study area. This may be caused by the structural flaws in the model, and/or the insufficient site-specific input parameters. So, to achieve good performance and reliable results of the WEPP model, more observation data is required from different ecosystems in Syria. These findings can provide guidance to planners and environmental engineers for proposing environmental protection and water resources management plans in the Coastal Region in Syria.

## 1 Introduction

The soil system is the cornerstone for the Earth system as it is the main path for different earth cycles (i.e. hydrological, geochemical, etc.) (Rodrigo-Comino et al., 2016). Thus, healthy soil is essential to reach the Sustainable Development Goals (UN-SDGs), in which soils play undeniable roles (i.e. Goals 2, 3, 6, 7, 12–15) (Keesstra et al. 2016, 2019). Among soil threats, soil water erosion is considered one of the main problems all over the world, and more than 80% of the world's agricultural soils are undergoing moderate to severe erosion (van Leeuwen et al., 2019; Nasir and Selvakumar 2018; Pimentel et al., 1995). Furthermore, soil erosion is considered the first enemy of sustainable agricultural practice, because it decreases agricultural productivity and crop yields by reducing soil quality (i.e. nutrient loss) (Guadie et al., 2020; Seitz et al., 2019, Mukanov et al., 2019; Tadesse et al., 2017). In addition, the eutrophication process takes place when the eroded soil reaches closed water bodies. Any recurrence of this process causes the accumulation of toxic and harmful substances in water bodies. Consequently, dissolved oxygen decreases in the water body.

Scientifically, soil erosion is the outcome of complex interactions among soil characteristics, land use, land cover, weather patterns, catchment area and topographical characteristics (Vanmaercke et al., 2011; Ziadat and Taimeh 2013). Water induced soil erosion is the final outcome of three stages, i.e. (1) the detachment of soil particles by the force of rain drops or overland flows, (2) transportation of the detached soil particles by runoff (Ellison, 1948; Sterpi 2003; Asadi et al., 2007; Shi et al., 2012) and (3) deposition in the downstream area. Soil erosion is considered one of the major natural hazards, which leads to land degradation all over the world. The process of soil erosion depends on three main agents, namely (1) rainfall (i.e. raindrop size and velocity, rainfall amount and intensity), (2) soil properties (i.e. aggregate soil stability; soil texture, density, and initial moisture), and, (3) local conditions (i.e. land cover, slope, micro topography, protection practices) (Salles and Poesen 2000; Issa et al., 2006; Dunne et al., 2010)., The interplay among these three key factors determines the erosion hazard in any region. The erosivity of rainfall and the erodibility of soil particles associated with local conditions affect the soil crust formation and runoff characteristics, which increase susceptibility to soil erosion (Issa et al., 2006). Nevertheless, soil aggregate stability performs a vital role against soil erosion. For instance, sandy soil has high detachability due to the domination of sand particles and an absence of cementing materials (i.e. clay; organic matter), while a more varied soil texture distribution can have low detachability. In addition, the critical shear stress of soil also plays a key role. Depending on the level at which a soil exceeds critical shear stress, several types of soil-water erosion can be distinguished (plan/inter-rill, rill, gully). The first is known as (plan) inter-rill erosion, when a selective transportation of fine particles occurs due to the inadequate capacity of inter-rill overland flow to transport large detached particles (Parsons et al., 1991), or to the selective deposition of coarse sediment (Proffitt and Rose, 1991). The second type of soil-water erosion is formed when the rill and gully erosion is less or non-selective for transporting soil particles (Shi et al., 2012).

The Mediterranean region is subjected to soil water erosion due to its typical climate regime, steep slopes (>25°), poor vegetation, soil compaction, and traditional tillage practices (Kosmas et al., 1997; García-Ruiz 2010; Rodrigo Comino et al., 2016; Rodrigo-Comino et al., 2017b; Keesstra et al., 2019). However, García-Ruiz et al. (2013) reported that soils in the Mediterranean region are the most fragile part of the ecosystems due to the slow soil formation process (1 t ha^−1^ yr^−1^), and the lower content of organic matter (≤2%), which threaten the biophysical environment and increase susceptibility to land degradation (i.e. soil water erosion). More recently, several strategies have been suggested to control soil erosion in agricultural lands, such as soil mulching (Cerdà et al., 2017), agri-spillways (Rodrigo-Comino et al., 2017a), catch crops (Kort et al., 1998), and soil terracing (Liu et al., 2013; Briak et al., 2019).

Since 1970, several mathematical models have been developed for estimating soil erosion, such as the Universal Soil Loss Equation model (USLE) (Wischmeier and Smith, 1965, 1978), and its improvement Revised Universal Soil Loss Equation (RUSLE) (Renard et al., 1997). Furthermore, previous literature suggests that the Water Erosion Prediction Project (WEPP) is one of the models designed primarily to predict soil erosion and runoff from hillslopes and small watersheds (Flanagan and Nearing, 1995, Flanagan et al., 2007). After extensive research in the field of soil erosion and conservation, American soil scientists developed the WEPP model (Nearing et al., 1989), which is regarded as a physically-based model (Grønsten and Lundekvam 2006; for more details, see Flanagan and Nearing, 1995). The WEPP model has been widely used around the world, including in India (Pandey et al., 2008; Singh et al., 2011), Italy (Pieri et al., 2007), Norway (Grønsten and Lundekvam, 2006), South Florida (Savabi, 2001), China (Zhang and Liu, 2005; Shen et al., 2009), Australia (Yu and Rosewell, 2001), the UK (Brazier et al., 2000) and Portugal (Vandekerckhove et al., 1998).

**Figure 1:**
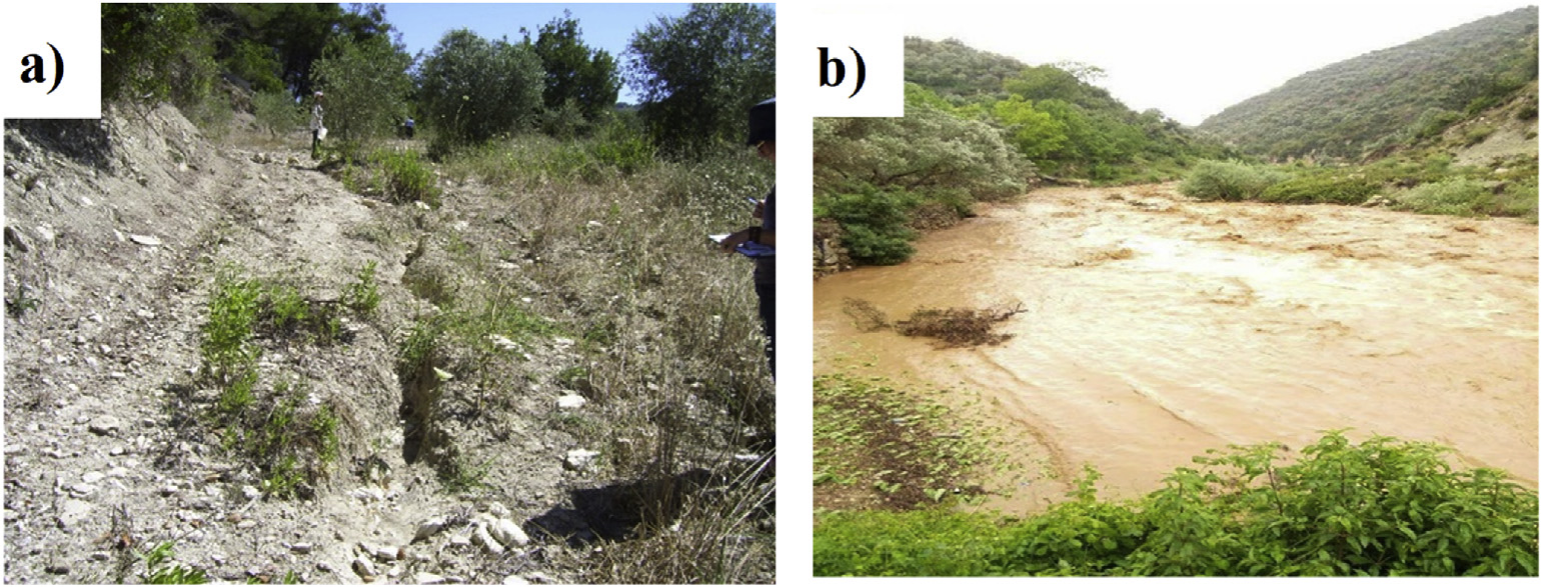
Soil erosion (a), and runoff (b) observed in the study area.

Since the 1990s, multifold studies have been conducted to address soil erosion in the Mediterranean basin. Unfortunately, a detailed spatial assessment of soil erosion in the eastern Mediterranean (Middle East) has not yet been carried out (Li and Fang, 2016). Syria, which is located in the Middle East region, has been affected by many environmental problems, such as soil salinization, desertification, overgrazing, cultivation in marginal areas, deforestation, and water and wind erosion, all of which threaten the sustainability of land resources. Furthermore, soil water erosion in the coastal area of Syria is a more prominent problem, as the coastal region is characterized by high soil erodibility, steep slopes, and high-intensity rainstorms events. Consequently, this problem affects agro-ecosystems (Figure 1) (Kbibo et al., 2017).

The literature survey showed that the previous studies on the evaluation of soil-water erosion in Syria did not consider any project monitoring systems. Rare studies were conducted using remote sensing technology and the Geographic Information System (GIS), although no validation nor accuracy assessments of the techniques applied were performed (Abdo and Salloum, 2017a, 2017b; Mohammed et al. 2016, 2020a; Barakat et al., 2013; Barkat et al. 2017). While a few studies have estimated the run-off and sediment yield in the coastal region of Syria using an empirical methodology (Mohammed et al., 2020b, 2020f, Kbibo et al., 2017; Kbibo et al. 2004), however, none have used an advanced modelling approach. Considering these research gaps, the present study aimed to use the advanced remote sensing based WEPP model to predict run-off for three different ecosystems in the coastal region of Syria. In addition, the present study attempted to verify the model performances for three different ecosystems by using several statistical techniques. Therefore, the novelty of the present study was the estimation and validation of run-off for three different ecosystems in the same study area using advanced modelling and statistical techniques.

## 2 Materials and methods

### 2.1 Study area

**Figure 2:**
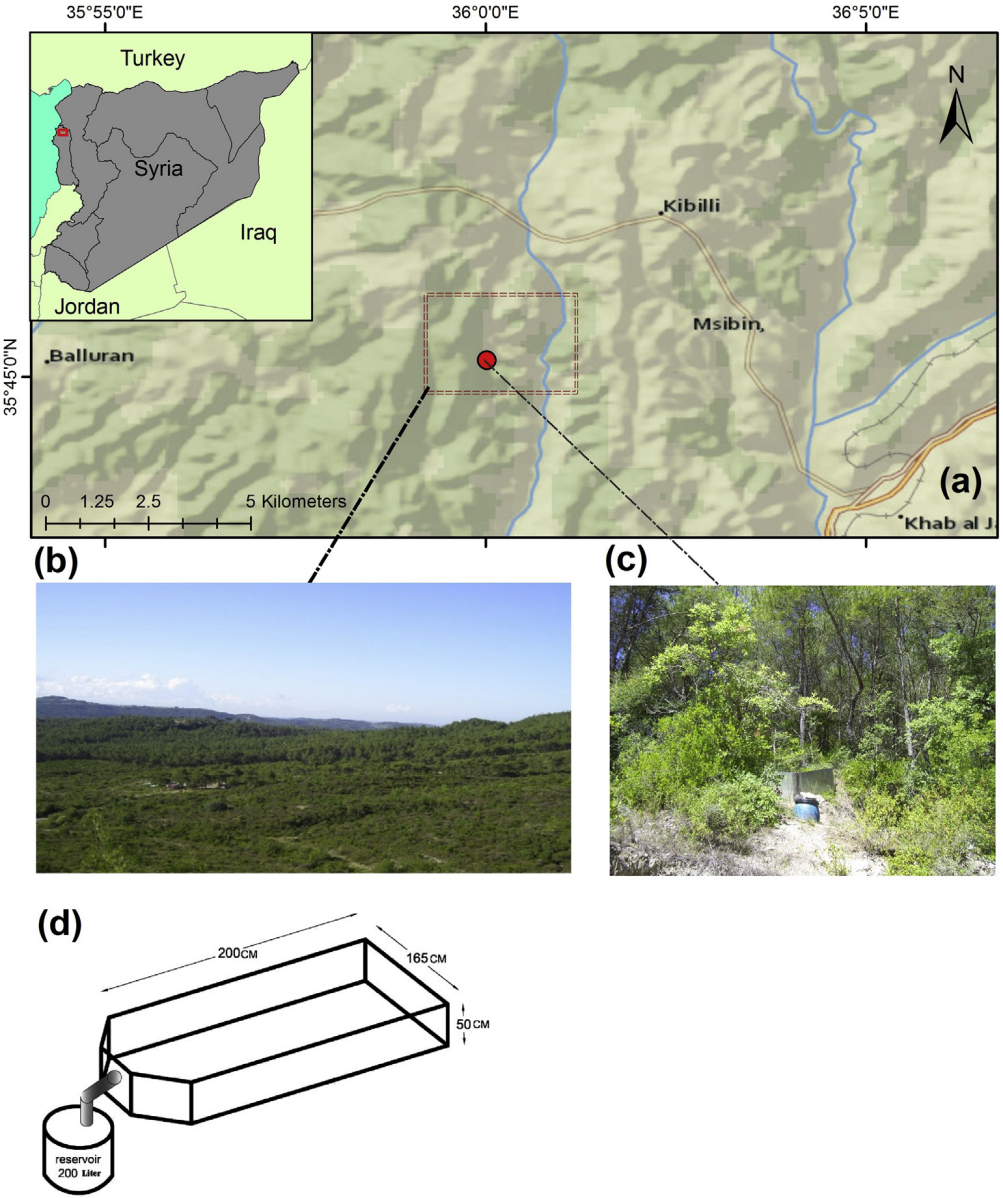
The study area and the experimental plot within the study area: a) the study area, b) an overview of the study area, c) one of the experimental plot set-ups, and d) sketch design.

This study was carried out in Al-Sabahia town (35° 45′ 14″ N, 36° 00′ 05″ E) in the northern Latakia governorate (W Syria) (Figure 2-a). The average elevation of the study area is 200m. The study area is dominated by the typical Mediterranean climate, which is characterized by a rainy winter and a dry summer. The average yearly temperature is 25 °C, and the average annual rainfall was about 750 mm over the period from 1990 to 2017 (Ministry of agriculture, 2018). The common soil type is Entisols (Mohammed et al., 2020c). Three types of land use/land cover can be found in the study area, i.e. forest, urban areas and agricultural farms. In addition, the study area is dominated by several of the most common crops, such as olives (*Olea Europea*), grapes (*Vitis sp*), figs (*Ficus Carica*) and walnuts (*Juglans Regia*).

### 2.2 Experimental set-up

After a field survey, three representative locations of land use within the study area were chosen. The first agro-ecosystem was the Agricultural Land (AG) (olive trees, three years old). Burned Forest (30% burned) (BF) with Pink rock-rose (*Cistus Creticus*) and Spiny broom (*Calicotome villosa*) formed the second agro-ecosystem, while the last agro-ecosystem was Forest (FO) (*Pinus brutia* and *Styrax officinalis*). From each location three soil samples were collected. Then, the samples were analyzed in a laboratory to determine the soil texture, the soil Organic Matter (OM) (%) (Nelson and Sommers, 1982), and the Cation-Exchange Capacity (CEC) (m.meq/100g soil) (Rhoades and Polemio, 1977), as shown in Table 1. Meanwhile, an experimental plot (2∗1.65∗0.5 m) was prepared to measure runoff and soil loss (Figure 2-b), while a rain gauge was placed next to each plot to measure precipitation. The experiment design had previously been adopted in the coastal and central part of Syria by Mohammed et al. (2020d). The run-off was measured after every rainstorm which exceeded 10 mm between 10/9/2012 and 10/12/2013. The amount of rainfall and the water that reached the reservoir were also recorded.

**Table 1:** Some characteristics of the soil in the locations studied.

	Clay	Silt	Sand	Texture	OM	CEC
	%				%	cmol_c_/kg soil
Agricultural land (AG)	34.1	47.3	18.6	Silty clay loam	1.7	41
Burned forest (BF)	40.1	39.4	20.5	Clay	2.29	44.5
Forest (FO)	31	51.6	17.4	Silty clay loam	5.2	36

OM: organic matter; CEC: cation exchange capacity.

### 2.3 Simulating runoff using the WEPP model

To calculate soil erosion, the model uses the continuity equation in a steady state (Nearing et al., 1989), as follows (eq. 1):

(1) $$\frac{\mathrm{dG}}{\mathrm{dx}}=\left(D_{f}+D_{i}\right)$$

where G is the sediment load (kg·s^−1^·m^−1^), D_i_ is the inter-rill sediment delivery to the rill (kg·s^−1^·m^−2^), x is the distance downslope (m), and D_f_ is the rill erosion rate (kg·s^−1^·m^−2^).

To simulate run-off, the Green-Ampt equation (Chu 1978) (eq. 2) was employed. This is expressed as follows:

(2) $$f_{\left(t\right)}=K_{e}\left(1+\frac{N_{s}}{F}\right)$$

where *f* is the infiltration rate (mm/h), K_e_ is the effective saturated hydraulic conductivity (mm/h), F is the cumulative infiltration depth (mm) and N_s_ is the effective capillary pressure (mm). The K_e_ plays a vital role in runoff by controlling the water infiltration, which is predicted by Zhang et al. (1995) (eq. 3):

(3) $$K_{e}=\;K_{b\;}\mathrm{TA}\;\left(1-SC_{\mathrm{ef}}\right)+\left(0.0534+0.01179\times K_{b\;}\right)P\times SC_{\mathrm{ef}}$$

$K_{b\text{:}\;}$ saturated (maximum) hydraulic conductivity (mm/h); TA: crusting and tillage factor. $SC_{\mathrm{ef}}$ : effective surface cover; P: precipitation (mm). For more details, see Zhang et al. (1996).

#### 2.3.1 Preparation of the input data for the WEPP model

This study used the WEPP (hillslope version), and the term “WEPP” hereinafter will be used to refer to the WEPP (hillslope version). To run the model, four types of input data, i.e. climate, soil, slope (topography), and management (land use/land cover) were obtained and prepared.

##### 2.3.1.1 Climate data

The CLIGEN-V5.3 model coupled with the WEPP model was used to generate climatic parameters. First, the file (NM-40016.GDS) was obtained from the USDA-ARS (http://hydrolab.arsusda.gov/nicks/nicks.htm). This data acted as the database for climate simulation)25 km from the studied area (. Subsequently, the CLIGEN model recommends that the Lahaina 3GL station (in the American state of Hawaii) acts as an alternative database for generating climatic parameters. Before considering a new climate station as a representative of the study area, some data was modified to prepare it as representative of the study area, including the maximum rainfall rate within 30 min, which was 48 mm, the maximum rainfall rate within 6 h, which was 111 mm, and the average yearly rainfall, which was 750 mm. The daily meteorological variables including the rainfall distribution, the temperature (maximum, minimum, and dew point), the solar radiation and the wind speed, were generated by the CLIGEN-V5.3 model which is considered the climatic input of the WEPP model.

##### 2.3.1.2 Slope files

The slope was prepared by the interface of the slope file builder. Then, the slope was set to 10% for 2∗1.65 m, which matched the experimental set-up conditions.

##### 2.3.1.3 Soil files

The data table of soil analysis was keyed to the soil input file. However, some data, including the effective hydraulic conductivity (mm/h), the rill erodibility (s/m), and the critical shear stress (Pa) were not measured, as we did not have the infrastructure to do this. To overcome these limitations, we calculated these parameters by the model itself, using the option “Have Model Calculated”.

##### 2.3.1.4 Management files

Management data was constructed by using the baseline file provided by the model. However, every ecosystem requires different management file options. For example, “Agriculture∖fallow – tilled” was used for AG, “Forest/30% Cover after Fire” was used for BF, and “Forest/Forest Perennial” was used for FO.

### 2.4 Calibration of the WEPP

Previously, the WEPP model had been calibrated for the Syrian coastal zone. The calibration process was performed by comparing the annual observed soil erosion data with the simulated soil erosion data obtained from 27 locations with different slopes and land uses. Interestingly, results showed that the predicted values were in good agreement with the observed values for different agricultural systems (R^2^ = 0.92, NSE = 0.84; RSR = 0.39; PBIAS = 13.05), and burned forest systems (R^2^ = 0.45, NSE = 0.34; RSR = 0.81; PBIAS = -12.01), but not for forest systems (for more details, see Mohammed et al., 2020d).

### 2.5 Statistical analysis and model performance

Box plots were used for plotting the simulated and observed run-off values (i.e. the minimum, maximum and medium) of each ecosystem by using the EViews program (McKenzie, and Takaoka. 2012). Some studied variables showed a non-normal or skewed distribution; therefore, the ANOVA (analysis of variance) test was employed for more than two groups together (i.e. AG., BF., and FO.). The Mann–Whitney test (Wilcoxon Rank Sum), which is a non-parametric test, was also used to compare every paired simulated and observed plot for each ecosystem (i.e. AG, BF, and FO). All statistical techniques were performed using the PAST software (version 4.01, https://folk.uio.no/ohammer/past/).

To evaluate the model performance, the linear regression model, Nash-Sutcliffe Efficiency index (NSE) (Nash and Sutcliffe, 1970), and the Standard Error of the Estimate (SEE) (Capra et al., 2009) were applied. The NSE ranges from -∞ to 1, and was calculated using Eq. (4):

(4) $$NSE=1-\frac{\sum {\left(y-0\right)}^{2}}{\sum {\left(0-\theta \right)}^{2}}$$

while the SEE was calculated by following Eq. (5):

(5) $$SEE=\sqrt{\frac{1}{n}}{\sum_{i=1}^{n}\left(0-y\right)}^{2}$$

where, y, 0 and θ are the predicted value, the observed value, and the average of the observed value, respectively. We also applied the Taylor diagram to visually evaluate the performance of the models. The Taylor diagram is a common plot of correlation (Pearson's r), the Root Mean Square Error (RMSE), and the standard deviation (SD). A good model has low model error (i.e. RMSE), is highly correlated with the observed values (the modelled values follow the observed value's pattern), and has an SD close to the dashed line starting from the observed value, indicating that the deviations around the mean are also in the right range (Taylor 2001).

## 3 Results

### 3.1 Daily observed soil erosion and runoff

**Figure 3:**
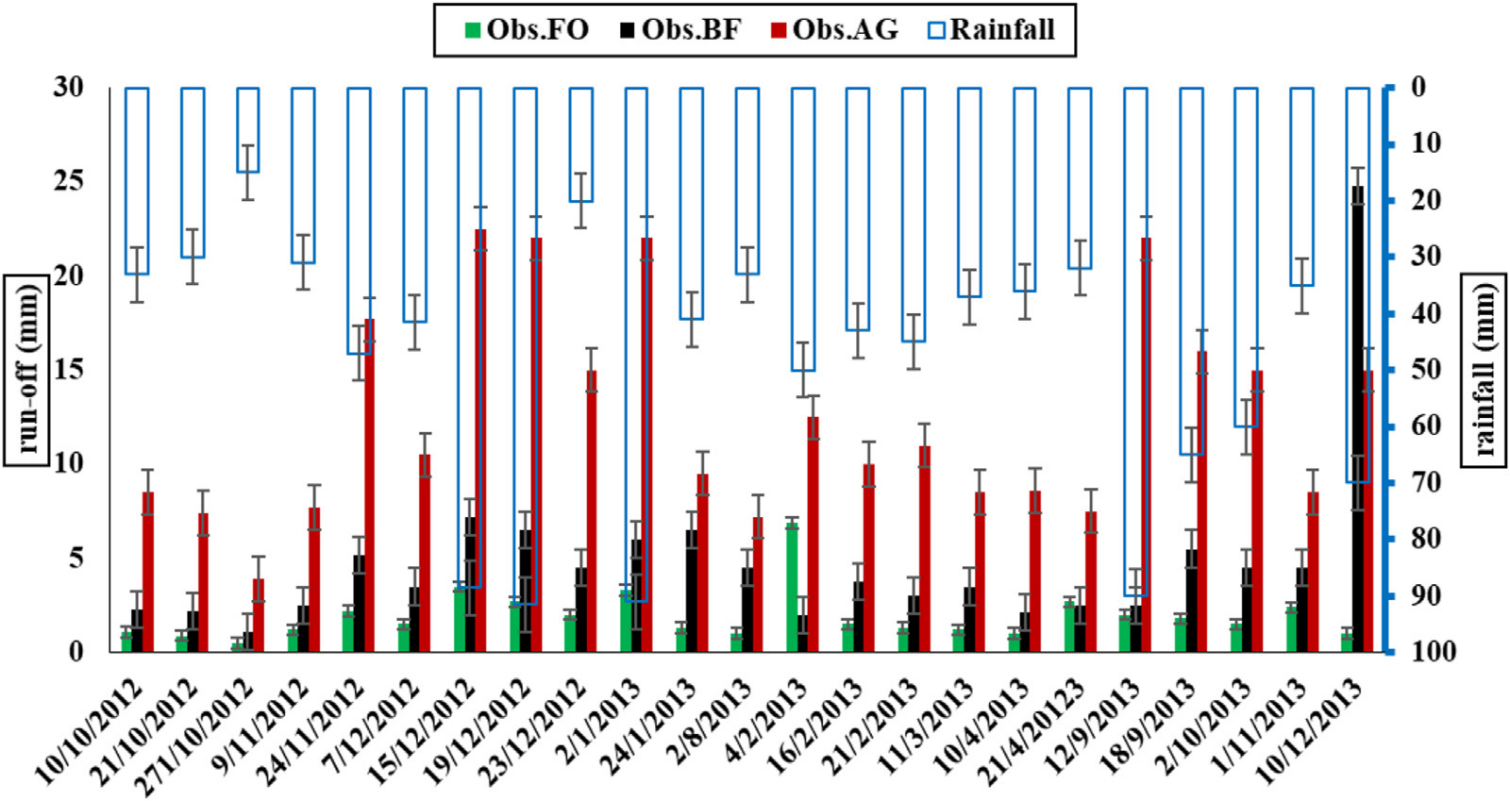
Runoff for the three ecosystems (filled bars) against daily rainfall values.

**Figure 4:**
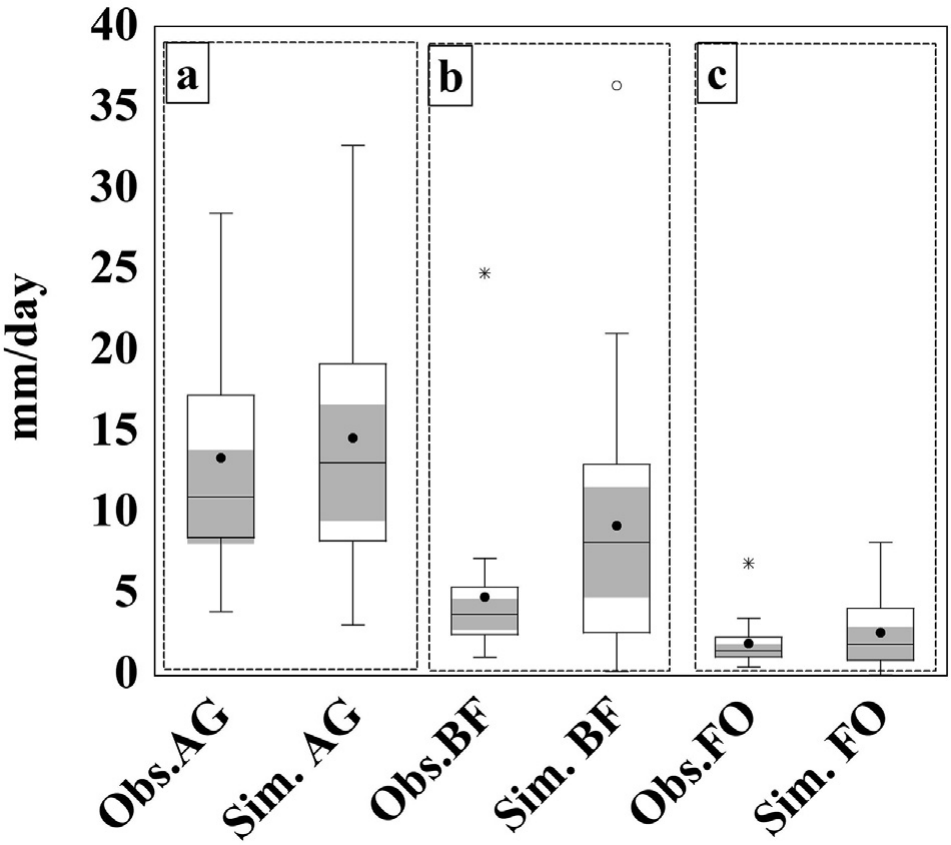
Box plots of simulated and observed runoff: a) AG: agricultural land, b) BF: burnt forest, and c) FO: forest. (median (^**_____**^); mean (•); near outlier (⁰); far outlier (∗), median 95% confidence (shaded)).

The observed runoff in agricultural ecosystems tended to have the highest value, followed by the BF and the FO, as shown in Figure 3. In addition, the highest observed runoff values were associated with the highest rainfall events in both December and January. However, in some cases, it could be noticed that even if the rainfall was not high, the observed runoff could have high values in agricultural land. For instance, the runoff from AG plots after events on 23/12/2012, 2/10/2013 and 1/11/2013 was 15mm; 15mm, and 8.5mm, while the rainfall was 20mm, 60mm, and 35mm, respectively. This type of unusual observation could be explained by the fact that the presence of soil saturation resulted from previous rainfall events. Furthermore, the measured run-off data showed that the average percentage of the runoff in the FO plot was 5% of the total precipitation, while it was 10.4% in the BF and 28.4% in the AG. This emphasized the vital role of land cover in preventing run-off. Moreover, Table 2 and Figure 4 showed the statistical analysis of the observed run-off data, where the average runoff was 12.54 ± 1.17mm/event in the AG, 4.81 ± 0.97 mm/event in the BF, and 1.72 ± 0.16 mm/event in the FO.

**Table 2:** Statistical analysis of observed data.

	Min	Max	Range	Median	Mean	σ	CV	SE
Obs.FO	0.50	3.50	3.00	1.50	1.72	0.77	0.45	0.16
Obs.BF	1.10	24.80	23.70	3.75	4.81	4.57	0.95	0.97
Obs.AG	3.90	22.50	18.60	10.50	12.54	5.48	0.44	1.17

Min: Minimum; Max: Maximum, σ Standard deviation; CV: Variation coefficient; SE: Standard error of the mean. Obs.FO: observed runoff from Forest (FO); Obs.BF: observed runoff from Burned forest (BF); Obs.AG: observed runoff from Agricultural land (AG).

### 3.2 Simulation of runoff by the WEPP model

**Figure 5:**
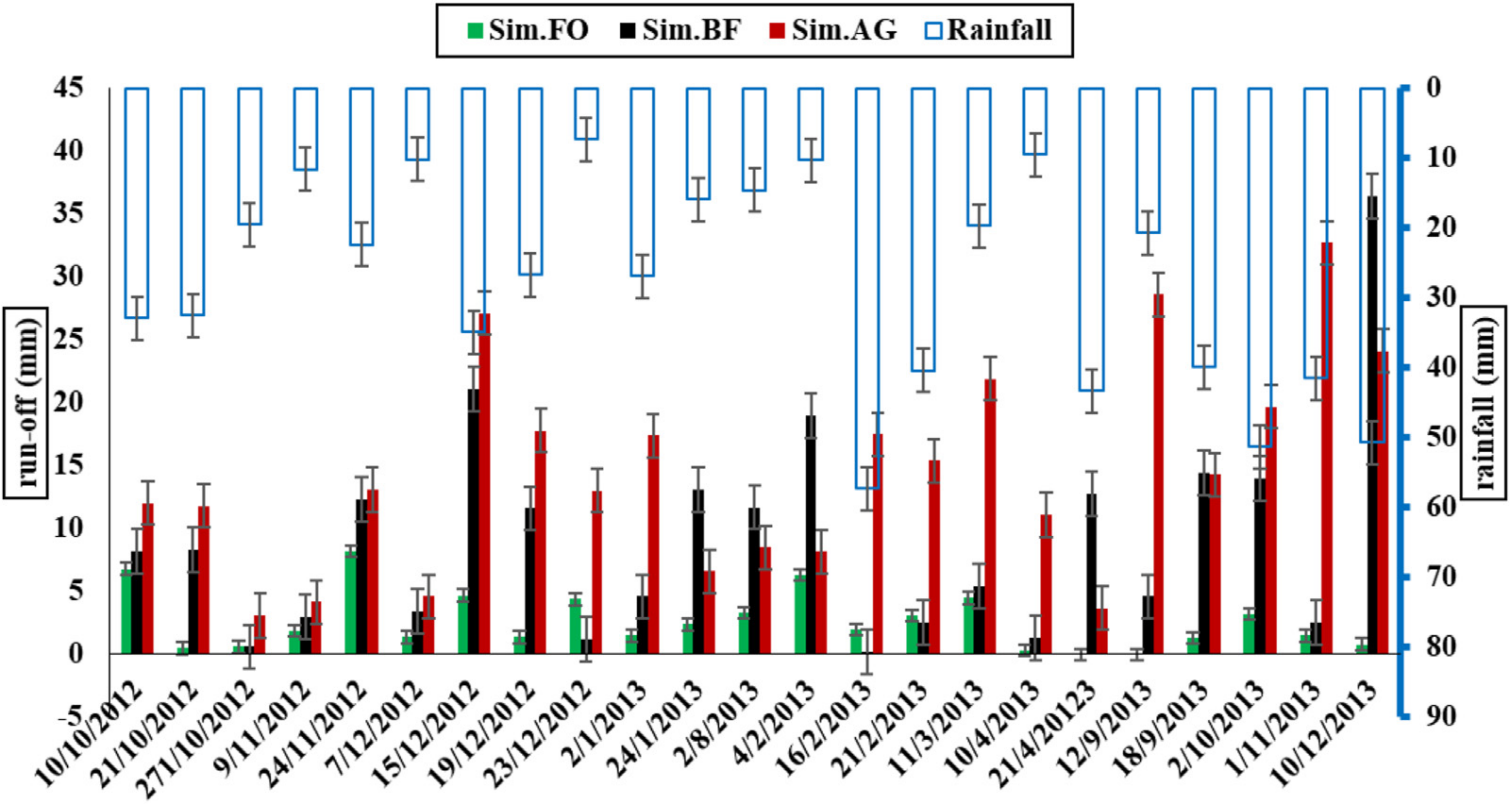
Event by event simulated runoff in the WEPP model.

The simulation results indicated that the WEPP model tended to produce more rainstorm events and runoff than the observed data. However, only the simulated events which were scheduled at the same time - i.e. at least in the same week as the observed events - were considered, regardless of the other generated events. Figure 5 illustrates that the highest runoff occurred in the AG, followed by the BF and the FO for most events considered. Nevertheless, the results showed that the runoff in the BF was higher than other ecosystems for some events, which cannot be true in reality. The results showed an error in the simulation output. The statistical analysis showed that the average runoff in the AG was 15.15 ± 0.89mm/event, while it was 9.23 ± 1.48mm/event in the BF and 2.61 ± 0.47mm/event in the FO (for detail see Table 3). Furthermore, Figure 5 demonstrated that the simulated values were higher than the observed values.

**Table 3:** Statistical analysis of simulated data.

	Min	Max	Range	Median	Mean	σ	CV	SE	SEE	NSE
AG	1.00	47.50	46.50	12.00	15.15	11.97	0.79	0.89	5.1	0.29
FO	0.00	8.20	8.20	1.90	2.61	2.22	0.85	0.47	6.9	-1.4
BF	0.20	36.40	36.20	8.20	9.23	8.29	0.90	1.48	2.2	-2.0

NSE: Nash-Sutcliffe Efficiency index; SEE: Standard Error of the Estimate.

### 3.3 Statistical analysis and evaluation of model performance

As illustrated in Table 4, the ANOVA test showed a significant statistical difference (p < .01) among the observed and simulated runoff values for different ecosystems, while the M-W test showed an absence of statistical differences between observed and simulated values, which indicated an identical median for each pair (i.e. obs. vs. sim.). In other words, the simulated run-off values resulting from the WEPP model were in agreement with the observed ones.

**Table 4:** Statistical analysis of runoff results.

	Ecosystem	Test	P-value	M-W *U*	z
Run-off Obs.	FO; AG; BF	ANOVA	1.10E-11∗∗	NA	NA
Run-off Sim.	FO; AG; BF	ANOVA	9.97E-07∗∗	NA	NA
Obs.AG vs. Sim.AG	AG	M-W	0.692	246	0.395
Obs. BF vs. Sim.BF	BF	M-W	0.073	182.5	1.792
Obs. FO vs. Sim.FO	FO	M-W	0.516	234.5	0.648

∗∗significant at the 0.01 level (two-tailed). Obs. Observed value; Sim.: simulated value; M-W = Mann–Whitney rank sum test, ANOVA = analysis of variance.

**Figure 6:**
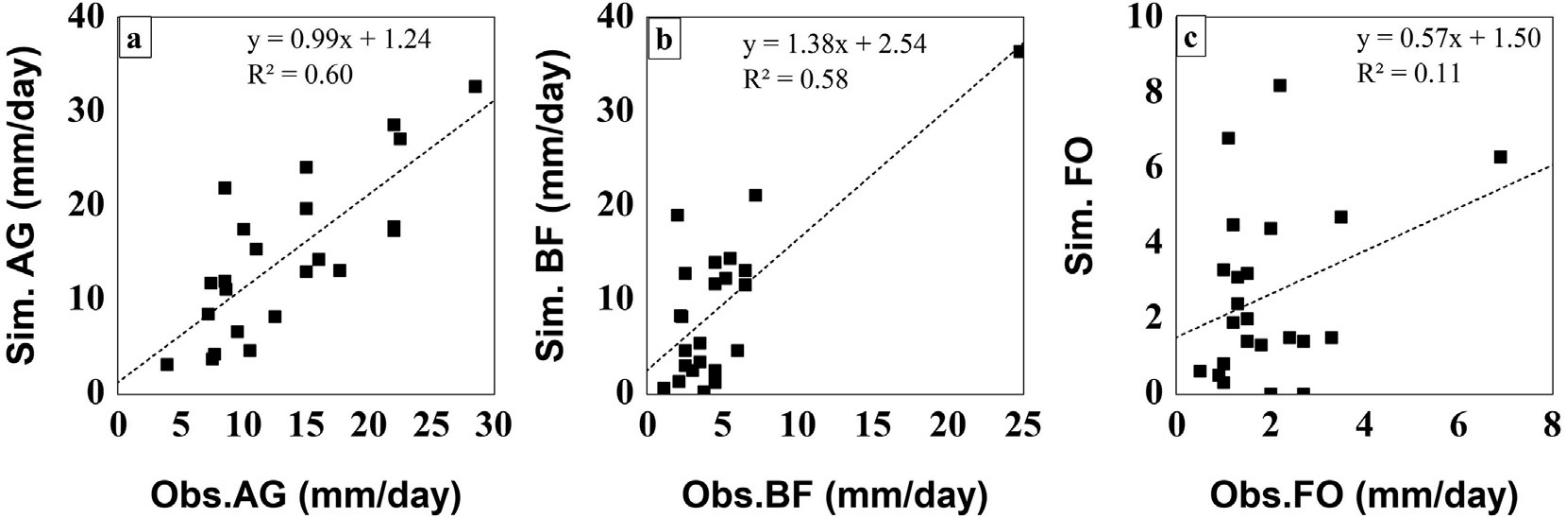
Correlation between simulated and observed runoff: a) AG: agricultural land, b) BF: burnt forest, and c) FO: forest.

**Figure 7:**
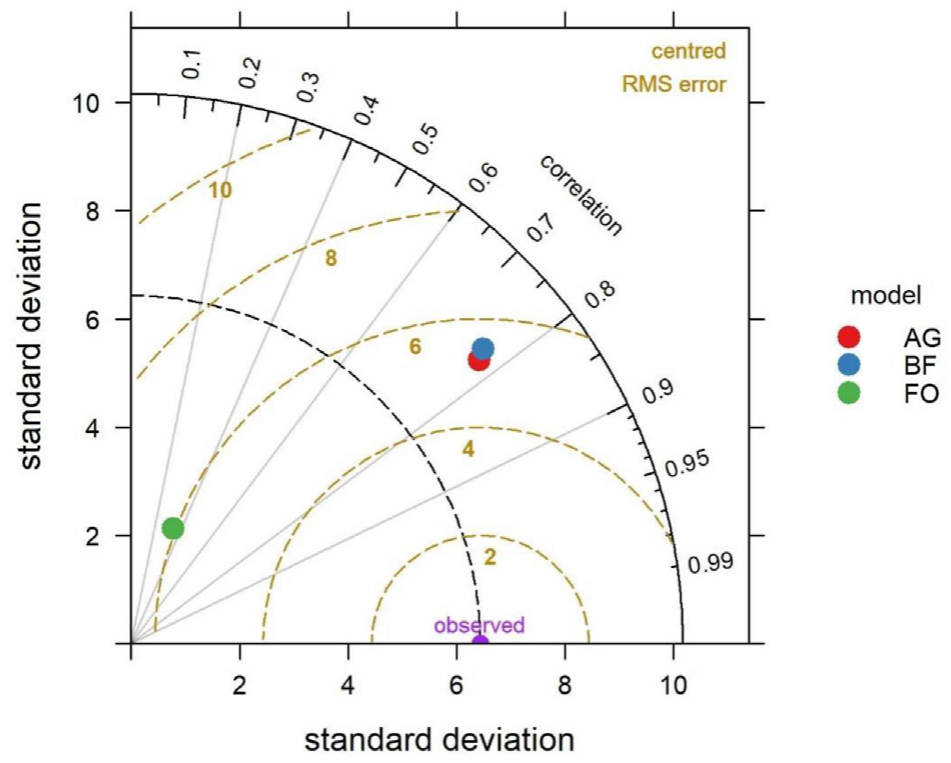
Model performance in predicting runoff using the Taylor diagram.

Figure 6 explicitly shows that the WEPP model had a better correspondence in the AG, followed by the BF and the FO. The degrees of collinearity between Sim and Obs values were expressed by the coefficient of determination (R^2^), which showed a good relationship (R^2^ higher than 0.5) between both the observed and the simulated values in both the AG and the BF (Figure 6). On the contrary, the results indicated a fair agreement between the observed and the simulated values of the FO, where the R^2^ did not exceed 0.11 (Figure 6). As can be seen from Table 3, the NSE and SEE values indicated a bad agreement between the observed and simulated values. It is obvious that the negative values of the NSE indicated a bad performance by the WEPP model. Similarly, the SEE value should be close to zero for a better performance. Obviously, the model performance was not satisfactory. The model performed best in the case of both the AG and BF models, and worst with the FO model. In the case of the AG and the BF the correlations were almost 0.8, the RMSE values were ~1 unit lower, and SDs were also closer to the observed values (Figure 7).

## 4 Discussion

In the Mediterranean part of Syria, the soil is exposed to water erosion due to conventional and non-conventional agricultural systems, inappropriate agricultural practices, overgrazing, deforestation, forest fires, and a lack of soil conservation strategies in cultivated areas. A similar situation can be found in other parts of the Mediterranean basin, including Spain (Rodrigo Comino et al. 2020; García-Ruiz 2010), Italy (Terranova et al., 2009; Novara et al., 2013), Portugal (Pastor et al., 2019), Lebanon (Kheir et al., 2008), Morocco (El Jazouli et al., 2017), Tunisia (Kefi et al., 2011; Kachouri et al., 2015) and Greece (Vantas et al., 2020). Thus, the modelling of soil erosion is one of the essential steps toward initiating soil conservation plans, especially in Syria, where the agricultural sector has been drastically affected by the ongoing conflict since 2011 (Mohammed et al., 2019). In addition, the testing and the validating of new erosion models, such as the WEPP model, which has never been applied in Syria, could provide an overview of WEPP performance under Mediterranean conditions, which are quite different from other places where the model was developed (USA). In the present study, we tested the WEPP model's applicability by using the run-off data measured from 9 field experimental plots (2∗1.65∗0.5 m), and the erosion/run-off data from the experimental plots are essential inputs for testing the model's performance in new testing areas (Bagarello and Ferro, 2004). To the best of authors' knowledge, the WEPP model was successfully applied for the first time to estimate daily run-off in the Mediterranean part of Syria.

### 4.1 Observed daily runoff

Scientifically, runoff and soil erosion are highly correlated with land cover, topography and soil properties (Nasir and Selvakumar 2018; Albaradeyia et al., 2011). The significant impact of three different types of soil management on the total runoff was observed in the study area. The AG system generated more runoff in comparison to the other two agro-ecosystems, regardless of the amount of rainfall. Identical findings have been found in the work of Ganasri and Ramesh (2016) reported that poorly managed agricultural activities, i.e. soil preparation and tillage, significantly increased soil erosion. The major difference between the runoff outputs could be caused by the different soil coverage, if other factors, such as slope, soil and rainfall intensity, are considered as constant. The vegetation coverage disperses the kinetic energy of rain and minimizes the direct effect of aggressive raindrops, which causes a reduced detachability of soil aggregates and slows down the runoff by minimizing the channel development (Roose 1996; Kefi et al., 2011).

Regardless of the fact that the observed rainfall events are not equal in terms of quantity, the observed runoff data reveals an obvious interaction among the soil, rainfall and land cover, where a general trend can be detected with the first rainy storms. Most of rainfall has been translated to infiltration, and no more than 20% of the total rainfall has been transformed into runoff. Furthermore, the continuous occurrence of rainfall can generate more run-off than the run-off generated from a discontinued occurrence of rainfall. For instance, the highest runoff was obtained on 23^rd^ December 2012, 2^nd^ October 2013, and 1^st^ November 2013. These events may be explained by the fact that the rainfall occurred again after a rainy day, then the soil became more saturated than on the previous day, as the soil surfacepores were already nearly saturated. Consequently, the extra rainfall is more likely to exceed the soil capacity of infiltration and finally generated the higher runoff. On the other hand, we observed an extreme rainfall event (>80 mm/day) which produced the highest runoff, which was expected to accelerate the soil erosion in the whole watershed. The impact of extreme rainfall events on soil water erosion and runoff in the Mediterranean region has already been highlighted by Ramos and Durán (2014), Cerdà et al. (2016).

Table 1 showed the soil properties of the study area. Most of the soils were characterized by the silty clay loam which had a good percentage of organic matter. Nevertheless, the highest OM was observed in the FO, resulting in a higher soil permeability in comparison with other studied plots, while it did not exceed 1.7 % in the AG, which increased the chance of soil runoff (Pimentel et al., 1995). In the Mediterranean region, good aggregate stability is one essential factor to combat soil erosion and runoff. In addition, good soil quality enhances aggregate stability against aggressive Mediterranean rainy storms, where microaggregate and macroaggregate stability depends on clay content and organic matter (Boix-Fayos et al., 2001); thus, low detachability could be expected. Also, the clay content and the OM directly impacted the amount of runoff by affecting the soil water content and hence the amount of runoff (Zakerinejad and Maerker, 2015).

### 4.2 Simulated daily runoff

The WEPP model predicted the runoff based on the difference between the rainfall and the infiltration rates by using the kinematic wave equations for a single event (Flanagan and Nearing, 1995; Amore et al., 2004). The WEPP model predicted 140, 126, and 90 runoff events for the ecosystems of the AG, the BF, and the FO, respectively. Generally, the land cover and vegetation cover play an important role in reducing runoff and soil loss by enhancing infiltration and decreasing soil erodibility, as well as improving soil quality (Shen et al., 2010). However, the findings of the simulation model showed that the lowest run-off in terms of events and amounts was predicted in the FO system, followed by the BF, and the AG (Figure 5). The WEPP model reflected the real situation successfully, with some exceptions on BF plots, where the model overpredicted runoff values (i.e. 9/12/2013; 10/2/2013). However, the findings of the M-W test indicated that there was no statistical difference between Observed and Simulated plots for each ecosystem (Table 4). Notably, the results from the Observed and Simulated plots exhibited almost similar behaviour when the predicted run-off of different ecosystems were compared with each other.

**Figure 8:**
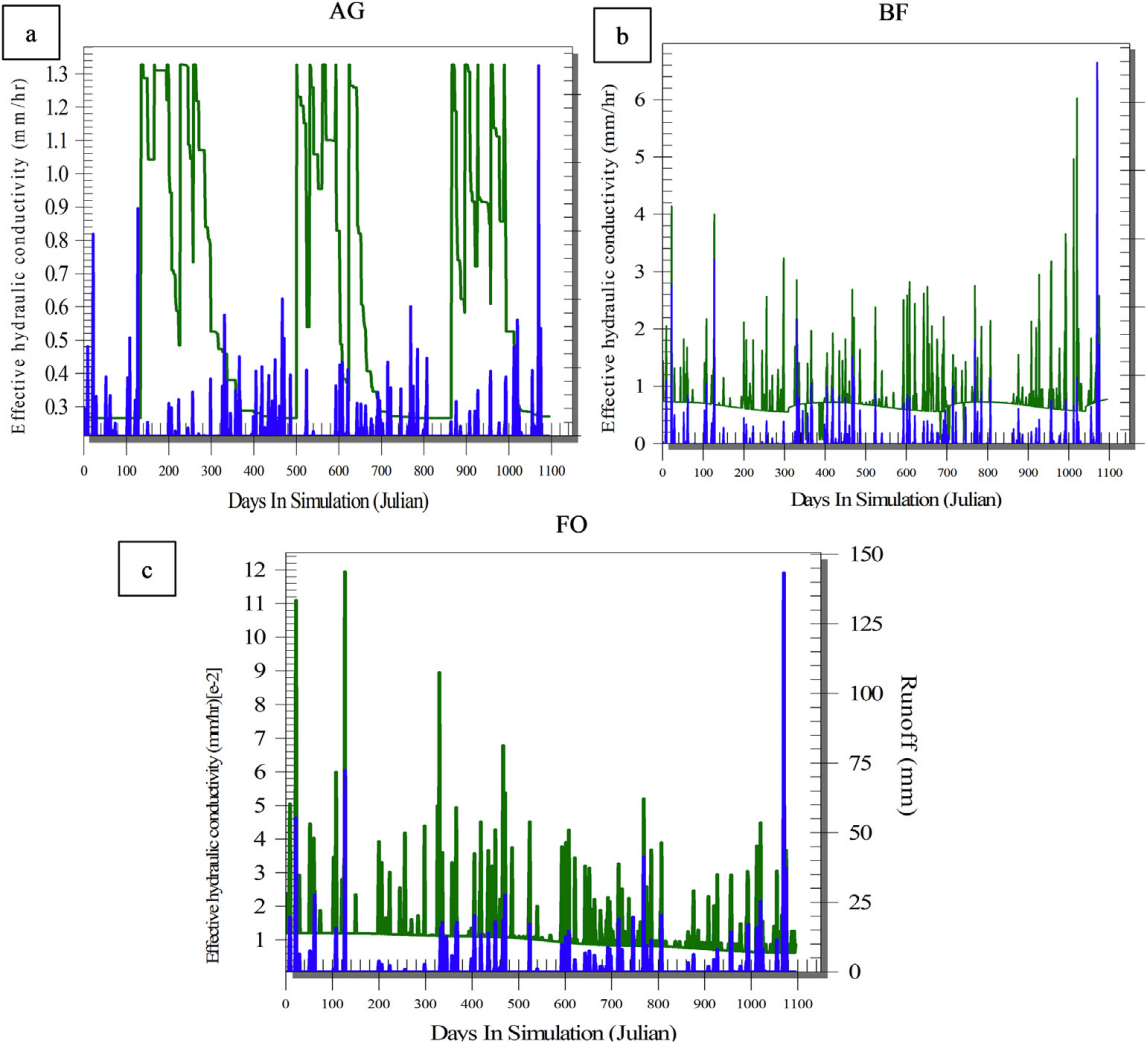
Runoff (blue line) and effective hydraulic conductivity (green line) in three ecosystems: a) AG; b) BF; c) FO.

**Figure 9:**
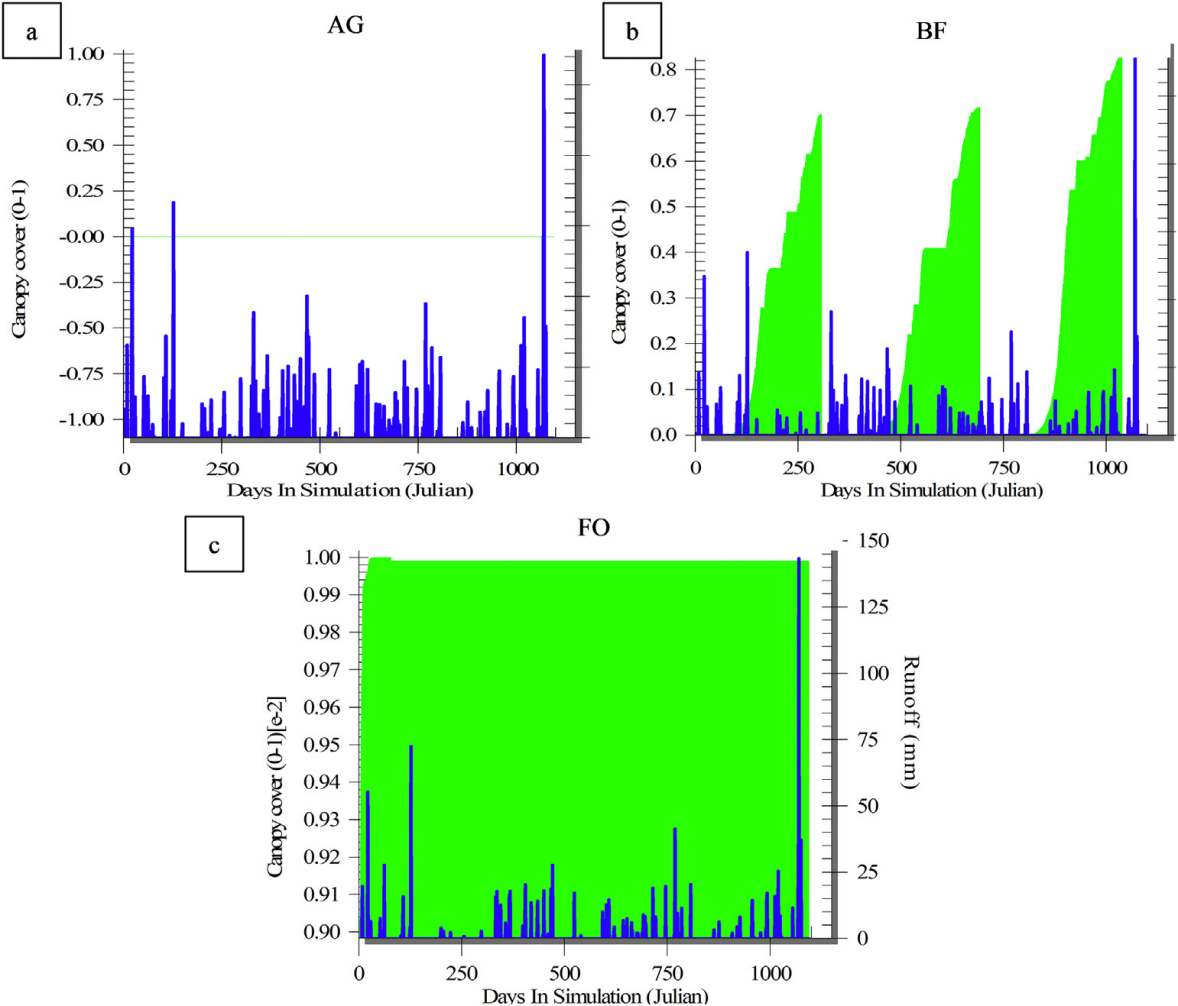
Runoff (blue line) and canopy cover in the three ecosystems: a) AG, b) BF and c) FO.

Tracking changes in effective hydraulic conductivity (K_e_) and runoff (Figure 8) showed that K_e_ in the AG plot (green line) did not exceed 1.6 mm/h, with remarkable seasonal changes, i.e. high in summer where the soil is dry, and low in winter where the soil is wet. This emphasized the fact that most of the predicted runoff occurred when K_e_ was low (Figure 8a). In addition, Figure 9-a showed that most of the runoff (blue line) occurred when there was no canopy cover. For both the BF and FO plots, the average K_e_ reached 8 and 12 mm/h, respectively (Figure 8-b and c) because of the presence of a high content of OM in the study area, which significantly increased the soil permeability and the water infiltration rate, as well (the green line). Figure 9-b and c showed that most of the predicted runoff occurred under the full land cover in the FO and partial protection in the BF. Interestingly, Figure 8-b strongly shows that the runoff rate (the blue line) was increasing, when the land cover (the green line) was moving toward the minimum level. In the plots studied, it was found that K_e_ increased in summer because of the drying and cracking of clayey soil, while K_e_ decreased in winter because of the wetness of the soil (Figure 8). Identical findings were reported by many researchers (Grønsten and Lundekvam 2006). Furthermore, Pandey et al. (2008) highlighted that the predicted runoff values were highly sensitive to K_e_. Based on these discussions, it can be concluded that the efficiency of the WEPP model was not satisfactory under current conditions.

Under the same inputs in climate, topography, and soil, with a different land management file, the WEPP model responded differently to each land cover. The high variability of the runoff prediction could be explained by the seasonal variation of the land coverage, which significantly impacted the model outputs. Similar findings were reported by Anache et al. (2018). Moreover, the average amount of runoff predicted by the WEPP model was higher than the observed value (Tables 2 and 3, and Figure 4).

Previous literature has already reported that the Green-Ampt/WEPP models have the tendency to overpredict the runoff for small events, while they underpredict the runoff for larger events (Risse et al., 1994; Ghidey and Alberts, 1996; Grønsten and Lundekvam 2006). Identical findings were found in the present study; i.e. that the simulated run-off values were higher than the observed run-off. These findings could be caused by the structural flaw of the model, and/or insufficient site-specific input parameters (Nyman et al., 2013; Risse et al., 1994; Mirzaee et al., 2017). As the main goal of this study was to evaluate the WEPP model's performance, no calibration had been undertaken for this particular location. Raclot and Albergel (2006) also applied the WEPP model to partially explain the disagreement between the observed and the simulated values. More recently, Kinnell (2017) reported that the WEPP model had the tendency to overestimate small runoff events and underestimate large runoff amounts, although the model was calibrated. However, the calibration of some soil characteristics could enhance the model performance in semi-arid and arid regions, as suggested by Mahmoodabadi and Cerdà (2013). However, many researchers have reported an accurate performance of the WEPP model in predicting runoff in various countries of the world, such as India (Pandey et al. 2008), China (Shen et al., 2010), Italy (Pieri et al., 2007), South-eastern Brazil (Anache et al., 2018), Iran (Akbari et al., 2015), and Spain (Soto and Díaz-Fierros 1998). Nevertheless, the findings of the present study are almost identical to those of the work of Raclot and Albergel (2006), which was conducted in the Mediterranean region (Tunisia).

One of the limitations of this study was an absence of certain data and parameters, such as inter-rill erodibility (K_ib_), rill erodibility (K_rb_), and critical shear stress (τ_cb_), which could enhance both the modelling process and the outputs. Such negative issues have been observed in different parts of the Middle East and North African (MENA) region, such as Iran (Amiri, 2010; Akhavan et al., 2010), Tunisia (Mtibaa et al., 2018), and many others. Also, the short period monitoring plots (two years) are inadequate to track the long-term variability of runoff, and to highlight the impact of extreme rainfall events, which have recently significantly increased in the coastal region of Syria.

Overall, the discussion has noted that the three different ecosystems responded differently to daily rainfall events and predicted different runoff values in the present study area. The highest runoff values were observed in agricultural plots, whether they were field measurement values or predicted by the WEPP model. This could be explained by the direct impact of anthropogenic activities such as tillage disturbance (Anache et al., 2018, Mohammed et al. 2020e), and trampling (Rodrigo Comino et al., 2016), which increase the susceptibility of soil aggregates to soil erosion and runoff. Meanwhile, the lowest observed and simulated runoff values were observed in the FO ecosystem. The findings of the present study, whether they were field measurement values or predicted by the WEPP model, had a good agreement with the fact that agricultural ecosystems can generate higher runoff and soil erosion than natural landscapes (Anache et al., 2017; Guo et al., 2015).

## 5 Conclusion

Daily runoff in the coastal area of Syria was measured using the experimental plots in three different ecosystems: 1) agricultural lands, 2) burned forest and 3) forest, to evaluate the performance of the WEPP model in predicting runoff in Syria. The final remarks can be surmised as follow:

1 The observed runoff data showed that the average runoff in agricultural plots was higher than in other plots, followed by burned forest, and then forest plots.
2 Similar to observed runoff data, the WEPP model prediction values showed that the average runoff in the AG, BF and FO were 15.15 ± 0.89, 9.23 ± 1.48 and 2.61 ± 0.47mm/event, respectively. However, simulated runoff values were higher than the observed run-off values.
3 The negative values of the NSE indicated a bad performance of the WEPP model compared with the observed data. However, the model performance was the best in the case of both the AG and BF models, and the worst for the FO model.

In this study, the efficiency of the WEPP model was not satisfactory, which can partially be explained by the fact that the input management files were used for modelling without any modification. In future research studies the input management files should be calibrated for each individual location. On the other hand, for an event-by-event simulation more research should be conducted to validate the model accuracy for future land degradation and conservation plans. Nonetheless, this research can provide the first insights about the application of the WEPP model in Syria. This study would provide insights for policymakers to solicit the adoption of new tools for predicting current and future soil erosion under different scenarios in the interests of soil and water conservation.

## Declarations

### Author contribution statement

Safwan Mohammed: Conceived and designed the experiments; Performed the experiments; Analyzed and interpreted the data; Wrote the paper.

Mais Hussien, Endre Harsanyi: Conceived and designed the experiments; Performed the experiments.

Karam Alsafadi: Analyzed and interpreted the data; Contributed reagents, materials, analysis tools or data.

Ali Mokhtar, Guido Rianna, Swapan Talukdar: Contributed reagents, materials, analysis tools or data.

Issa Kbibo, Mona Barkat: Conceived and designed the experiments; Performed the experiments.

Szilárd Szabó: Analyzed and interpreted the data.

### Funding statement

Open access funding provided by University of Debrecen. Szabo, S. was supported by the project TKP2020-IKA-04, implemented with the support provided from the National Research, Development and Innovation Fund of Hungary, financed under the 2020-4.1.1-TKP2020 funding scheme.

### Data availability statement

Data will be made available on request.

### Declaration of interests statement

The authors declare no conflict of interest.

### Additional information

No additional information is available for this paper.
